# Artificial Intelligence Driven Framework for the Design and Development of Next-Generation Avian Viral Vaccines

**DOI:** 10.3390/microorganisms13102361

**Published:** 2025-10-14

**Authors:** Muddapuram Deeksha Goud, Elisa Ramos, Abid Ullah Shah, Maged Gomaa Hemida

**Affiliations:** Department of Veterinary Biomedical Sciences, College of Veterinary Medicine, Long Island University, Brookville, NY 11548, USA; muddapuramdeeksha.goud@my.liu.edu (M.D.G.); elisa.ramos2@my.liu.edu (E.R.); abidullah.shah@liu.edu (A.U.S.)

**Keywords:** artificial intelligence (AI), epitope prediction, immunoinformatics, AlphaFold2, in silico immune simulation, machine learning (ML)

## Abstract

The rapid emergence and evolution of avian viral pathogens present a major challenge to global poultry health and food security. Traditional vaccine development is often slow, costly, and limited by antigenic diversity. In this study, we present a comprehensive artificial intelligence (AI)-driven pipeline for the rational design, modeling, and optimization of multi-epitope vaccines targeting economically important RNA and DNA viruses affecting poultry, including H5N1, NDV, IBV, IBDV, CAV, and FPV. We utilized advanced machine learning and deep learning tools for epitope prediction, antigenicity assessment, and structural modeling (via AlphaFold2), and codon optimization. B-cell and T-cell epitopes were selected based on binding affinity, conservation, and immunogenicity, while adjuvants and linker sequences enhanced construct stability and immune response. In silico immune simulations forecasted robust humoral and cellular responses, including cytokine production and memory cell activation. The study also highlights challenges such as data quality, model interpretability, and ethical considerations. Our work demonstrates the transformative potential of AI in veterinary vaccinology and offers a scalable model for rapid, data-driven vaccine development against avian diseases.

## 1. Introduction

### 1.1. An Overview of Artificial Intelligence (AI)

**Figure 1:**
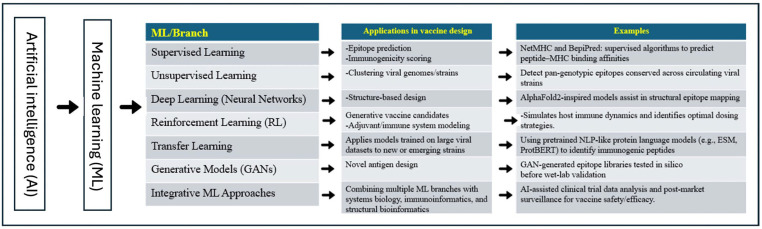
A schematic representation chart showing the applications of the AI tools, particularly ML branches, in the design and development of the next generation vaccines against common viral diseases of birds.

Artificial Intelligence (AI) refers to technology that simulates human intelligence, including language recognition and translation, visual perception, and problem-solving, to enable computer systems and machines to perform similar tasks in a remarkably short time with great precision. AI is continuously evolving with improved algorithms and statistical data to provide technological advancements in various areas, ranging from automotive and media to healthcare. AI has significantly evolved since its first introduction in 1955. Since then, AI has evolved from utilizing historical data, as in machine learning (ML), to deep learning, which explores more complex patterns to build layers of neural networks of information. As technology evolves rapidly, a newer form of AI, called “Generative AI,” emerges [1]. Based on collections of training data, it can generate new text, images, and/or audio. Generative AI has a greater focus on information technology, expanding into areas such as education, research, and marketing, compared to deep learning, which has had a significant impact on health systems [2,3]. Our main objectives are to highlight the roles of AI and its branches in the advancement of various aspects of vaccine design and development for the major poultry viral diseases (Figure 1). We also emphasized the mandate of integrating the AI tools supported by the functional analysis to speed up the veterinary vaccine pipelines, particularly for the emerging/reemerging viral diseases of poultry.

### 1.2. Current Challenges in Veterinary AI Adoption

The benefits of AI seen in human healthcare are also seen in the field of veterinary medicine. The rising need for veterinarians and the increased demand for veterinary services have put immense pressure on current practices to maintain the gold standard of care while also maintaining a positive working environment for hospital staff [4]. AI has many clinical applications in the field of veterinary medicine, such as improving the image analysis in radiology or MRI, or aiding areas of production animal medicine [4]. AI could also enhance the design of some novel vaccines and antimicrobial drugs [5,6,7,8]. It has also been recently used in the identification of some novel receptors or enzymes required for some viral replication, particularly coronaviruses [9].

### 1.3. AI Applications in Veterinary Medicine

In clinical practice, client communications are key to gathering pertinent information to develop a care plan and generate detailed client records. Invaluable details can be missed between the conversation in the exam room and writing a complete SOAP note. One AI algorithm, Cluster2Sent, has been found to extract important information from recorded physician-client communications, cluster related pieces of information, and then generate summaries based on the clusters [10]. This algorithm reduces the burden upon veterinarians and allows for more detail to be included in patient records, giving the client clear information on the visit and the physician’s security, as SOAP notes can be used during miscommunications or legal discussions [11]. There are many applications of AI and its branches in the field of veterinary medicine, including drug design and repurposing of some known drugs for new targets, screening large libraries of compounds as potential new therapies for many diseases of animals and birds, and enhancing the design of next-generation vaccines for animals and birds, studying the viral evolution and the mechanisms of emerging new viral diseases [5,7,8,9,12,13,14], helping in the diagnosis of many animal diseases and improving the quality of the currently used diagnostic assays, including MRI, CT scans, etc. [15,16,17,18]. The AI and its tools will revolutionize many research directions and the applications in veterinary medicine in the near future.

### 1.4. Some Potential Roles of AI/ML in Studying Viral Diseases in the Context of the One Health Concept

In the frame of the One Health initiative, AI can be used to understand and mitigate the massive impact of zoonotic diseases using datasets such as environmental factors, genetic sequences, and public health records in machine learning models and advanced algorithms [19]. Rift Valley Fever (RVF) causes massive die-offs in livestock, but the occurrence and factors for transmission are not clear. Using neural network techniques, an epidemiological model of RVF was developed using landscape, climate, and historical data to better understand the influential factors of disease as the threat of infection increases with climate change [19]. Further development has occurred with various infectious diseases using AI, such as epitope predictions, immune stimulation, and more, to generate various vaccines and peptides (Table 1). Most of the viruses that are detailed in Table 1 have animal hosts, but humans can still be significantly impacted both directly and indirectly. Virology encompasses both humans and animals, and with the aid of AI, there can be a deeper understanding of infectious diseases that are emerging or re-emerging, leading to more effective future solutions.

### 1.5. Accelerating Vaccine and Antiviral Therapy Development Using the Enhanced ML/AI Tools

Previously, the development of vaccines and antiviral therapies could take years and was expensive, not just to produce but also to research [56]. Key struggles in the previous methods of vaccine manufacturing and research did not address antigenic diversity, which is a challenge for complex pathogens [56]. With the use of enhanced ML/AI, complexities can be analyzed by various algorithms to identify novel antigens and predict antigenic epitopes, and other diverse features of pathogens in a shorter amount of time and with fewer resources required [56]. The rapid growth in genomic research from the use of artificial intelligence allows researchers to make discoveries and improve the durability and potency of vaccines [57]. Vaccine development, even with AI, still requires funds and resources, but an AI tool can be used to predict vaccine safety and efficacy, so that resources are directed to better quality research candidates [57]. To further accelerate antigen discovery and epitope optimization, AI tools such as AlphaFold2 and NetMHCpan integrate deep learning models, immunological data, and population-scale genomic data. Similarly, in antiviral therapies, viruses continue to evolve, and that results in the emergence of drug resistance [58]. Innovative AI technologies are required to accelerate the timeline for new therapies [58]. Platforms that drive AI drug discovery rapidly identify and optimize antiviral compounds. These platforms analyze drug-target interactions, along with predicting efficacy [59]. These innovations do contribute in a collective way so that humans and animals can control viral diseases more effectively.

### 1.6. Integration of AI in Vaccine Design Enhances the Pandemic Preparedness Programs in the Context of One Health

Several emerging and reemerging viral diseases have pandemic potential, particularly influenza and coronaviruses [17,60,61,62,63,64,65]. Continuous monitoring of viral diseases through the ongoing molecular and serological surveillance, then analyzing the obtained sequences for each virus using the bioinformatic analysis tools will help us in monitoring the emerging and reemerging viral diseases on the genomic levels. This approach will help us in upgrading the currently used vaccines and diagnostic assays to match the currently circulating strains/variants/clades of these viruses in the field [61,63,64,66]. This approach will make us much more vigilant against any substantial change in these emerging and reemerging viral diseases; thus, the risk of any upcoming pandemic could be mitigated. Application of AI tools, particularly various branches of ML, will have several advantages for vaccine design and development of the next generation vaccines compared to conventional vaccines. The AI tools and the ML branches will speed up and expedite the vaccine development pipelines, and they will also increase the precision of the vaccine design. This is in addition to the selection of the right adjuvant that could potentiate the actions of the designed vaccine.

## 2. Historical and Conceptual Background of Vaccines and the Integration of AI in Modern Vaccine Development

### 2.1. Types of Currently Known Vaccines

Vaccines vary based on the evolution of methods used to produce them and specific vaccine characteristics, such as the spectrum of effectiveness. The term “generation” is used to divide and categorize the vaccines that have been developed over time. Specifically, there are three generations of vaccines. The generations of vaccines are divided based on the technology used to develop the vaccines, such as direct exposure to a virulent virus, which is considered first generation, whereas second-generation vaccines utilize non-pathogenic components of the virus. The third generation of vaccines utilizes a genome-based technology focusing on various forms of recombinant DNA and possible optimizations with AI [67].

#### 2.1.1. First Generation Vaccines

Due to the primary methods of development, this generation is more associated with conventional vaccines [68].

**Live attenuated vaccines**: Like the polio and measles vaccines, these were developed by weakening the virus of interest to still stimulate the immune response and produce memory cells, but cause no or mild disease. The virus is passed through an unnatural host, leading to adaptive mutations. The mutations do not affect the growth of the virus in the body but cause the virus to lose its virulence. However, there are side effects that can occur. The virus could revert to virulence, causing the disease to develop in the host post-injection [67].

**Heterologous viral vaccines:** These vaccines utilize viruses that are antigenically related to each other. Then, protection arises from the presence of cross-reacting antigens. In veterinary medicine, an example is the use of the rinderpest virus and measles virus to protect against the canine distemper virus [67].

**Inactivated viral (attenuated) vaccines:** From the process developed by Louis Pasteur, for these vaccines, the virus is propagated using physical methods, such as formalin, heat, or chemical methods, such as UV, to inactivate the virus. This process kills the organism without changing the antigenic structure, causing the virus replication to be inactivated. These vaccines are expensive to produce and often require an adjuvant to enhance the magnitude of the host’s immune response [67].

#### 2.1.2. Second-Generation Vaccines

In the first generation of vaccines, there was a possibility that the virus could revert to virulent infection in the host [68]. The next generation utilized elements in the viral genome, such as proteins or non-protein antigens, to produce vaccines [68].

**Subunit vaccines:** Using viral components such as the capsid or proteins, a vaccine is prepared that is devoid of viral nucleic acids to induce an immune response without febrile reactions. These vaccines are safer and highly immunogenic in high doses. There are various types of subunit vaccines, such as those using a replicon, viral vector, or plasmid that is expressed in bacteria. For Feline Leukemia Virus, the vaccine utilizes a feline oncornavirus cell membrane-associated antigen (FOCMA) for viral glycoprotein 70 [67].

**Genetically engineered vaccines:** With newer technologies, there has been progress in manipulating the nucleotides in DNA/RNA viruses, utilizing a plasmid to be delivered through injection [69]. Antigens of interest encoded in the vaccine are expressed using the host cell’s machinery to elicit an immune response [69]. The DNA viral genomes, like those in African swine fever, are easier to manipulate than RNA genomes with the genetic engineering approaches [70]. For RNA Viruses, the viral live mutant, infectious clones are added to a plasmid, and in bacteria, the virus is propagated to be utilized in a vaccine. Differently, in DNA viruses, they can be altered by deleting a gene to allow replication, deleting a gene to make replication defective, or a version of a subunit vaccine by altering a single gene [67].

#### 2.1.3. Third Generation Vaccines

Genetic immunization utilizes a plasmid carrying an encoded antigen. Any vaccine that involves DNA or RNA genetic immunization is considered within this generation [68].

**DNA Vaccines:** The plasmid DNA is introduced into the host cells. The vaccine is administered intramuscularly using a Gene gun [71]. The antigen-presenting cells in the host internalize the plasmid to then integrate the DNA into the host cell genome. The antigen is then synthesized in the host to produce an immune response utilizing MHC I and II cells and Th1 cells [67].

These various vaccines have improved as technology has become more available, and there have been more breakthroughs in science. However, generally, these vaccines tend to take a long time to make and can be expensive. With so much more technology, such as artificial intelligence, vaccine development and design can become more efficient and beneficial to researchers and citizens to prevent infectious diseases.

### 2.2. Integration of ML/AI and Bioinformatics in Vaccine Design and Evaluation

The initial idea of developing a vaccine seems straightforward until the reality of development sets in. Artificial intelligence is able to learn algorithms and interpret viral genetic information to identify key targets within the virus [72]. New emerging pathogens and increased antimicrobial resistance have increased the use of AI-assistance in vaccine development and design [73]. One technique that has revolutionized vaccine development is Reverse Vaccinology, or RV [73]. RV focuses on a genome-based approach to vaccine development that uses computational bioinformatics to screen for potential vaccine candidates [73]. When incorporating AI technologies, vaccine candidates are optimized, and the time it takes for the discovery of candidates is expedited [73]. AI prediction models, such as Vaxijen, identify antigens that stimulate the immune response, the first step in vaccine development [73]. Based on learned information, the Vaxijen system assumes that antigenicity is coded within the protein sequences, specifically, the chemical properties of the amino acid residues [73]. Focusing on poultry vaccines, an AI tool was used to identify the affinity of binding to MHC class 1 allele to identify the peptide in as many Newcastle Disease Virus (NDV) sequences, but filter out proteins with the potential to produce an autoimmune response [31]. The AI/ML tools can analyze data using various algorithms and verify the accuracy before applying that knowledge to outperform pathologists in identifying biomarkers or expedite tissue sample evaluation [74]. AI is an effectively employed tool that aids in further expanding the modern age of vaccine development.

## 3. AI-Driven Approaches in Vaccine Design and Development

### 3.1. Comparative Analysis of AI/ML and the Traditional Approaches in Vaccine Design and Development

Traditional vaccine development is heavily dependent on supplies, time, and money. Conventional diagnostic methods rely on laboratory facilities and trained staff [75]. To confirm the infections, like in Avian influenza, considerable time is needed, but early identification is key in stopping the spread of the virus [75]. The spread and losses caused by highly pathogenic Avian influenza can lead to extreme economic losses, like those seen in 2014, due to a five-day lag between initial signs and quarantine [75]. Applications of more advanced technology will allow for increased disease diagnosis to reduce the level of potential hazards to both humans and animals [75]. The application of AI tools could lead to a substantial decrease in the timeline required to identify targets, streamline vaccine candidate development, and expedite the development of future vaccines [74]. In traditional methods, the product is not guaranteed to produce results, but with the use of AI, key components for vaccine development are provided with greater accuracy and consideration to develop better vaccines [72].

### 3.2. Identification of the Key Viral Proteins/Immunogens for Vaccine Design

The first and most critical step in vaccine development is the identification of potential immunogenic targets, followed by the rigorous discovery of antigens. In birds, viral pathogens—particularly RNA and DNA viruses—pose a chronic risk to global food security and economic stability worldwide. Classical approaches to antigen discovery, including attenuation or inactivation of whole pathogens and empirical testing of protein fractions, are limited by high costs, extended time requirements, and reduced efficacy when faced with rapidly mutating viruses [76]. The development of artificial intelligence (AI) and its branches, including machine learning (ML) and deep learning (DL), has significantly accelerated the process of identifying the right target proteins/immunogens for each virus [77].

Poultry are afflicted with a variety of high-priority RNA viruses, including H5N1 avian influenza virus, infectious bronchitis virus (IBV), Newcastle disease virus (NDV), avian metapneumovirus, avian encephalomyelitis virus, avian leukosis virus, and infectious bursal disease virus (IBDV). These pathogens mainly display surface or capsid proteins like HA, S1, F, G, VP1, gp85, and VP2, which are primary immunological targets because they directly interact with host immune surveillance systems [78,79]. DNA viruses like chicken anemia virus (CAV) and fowl pox virus (FPV) also have significant effects on poultry health, and proteins like VP1 and VP4 play critical roles in capsid assembly and immunogenicity [80,81].

The initial step of AI-assisted target identification involves the retrieval of protein sequences from databases like NCBI GenBank and UniProt. For instance, the H5N1 HA protein (Acc. No. AAR02643.1), IBV S1 (AAT62301.1), and NDV F protein (NP_056739.1) were retrieved and cross-referenced for annotation using BLASTp and InterProScan, to validate structural motifs and immunologically dominant domains [82,83]. Proteins were prioritized based on key selection criteria: surface expression, antigenicity, absence of homology to avian host proteins, and sequence conservation among different serotypes or genotypes [84].

The use of AlphaFold2 for such proteins is a significant advancement in understanding antigenic topography. By accurately predicting the three-dimensional structures of viral proteins, AlphaFold2 has enabled the visualization of conformational epitopes that are hidden when only linear sequences are considered [85,86]. Validation of such models was carried out using pLDDT confidence scores and Ramachandran plots, which allowed for the recognition of surface-exposed loops and areas of high epitope potential [87]. For example, conformational epitopes in the HA stem region of H5N1 and the S1 domain of IBV were mapped to conserved β-sheet areas that are exposed on the virion surface [78,87]. To predict the B-cell epitopes, BepiPred 2.0, ABCPred, and DiscoTope algorithms were used to detect linear and discontinuous epitopes from amino acid characteristics like hydrophilicity, flexibility, and surface accessibility [88,89]. These epitopes play an essential role in the humoral immune response through antibody recognition and neutralization. B-cell epitope mapping was particularly revealing in IBDV-VP2 and CAV-VP1 proteins, where surface-exposed loops and variable domains are immunodominant [80].

To induce T-cell-mediated immunity, CD8+ and CD4+ epitopes were predicted by employing the NetMHCpan and the NetMHCIIpan tools. These platforms predict MHC-peptide binding based on position-specific scoring matrices and neural network models trained using known binding data [90,91]. The major histocompatibility complex (MHC) allele BF2*21 has been well characterized in chickens, and the application of AI tools facilitates the precise identification of high-affinity peptides with the ability to induce potent cytotoxic and helper T-cell responses [92]. High-binding affinity (IC50 < 50 nM), epitope conservancy, and lack of cross-reactivity with self-proteins were employed as strict filters for selection [93,94].

The immunogenic epitopes were divided into linear B-cell, conformational B-cell, CD8+ cytotoxic T-cell, and CD4+ helper T-cell epitopes. Only those epitopes that were predicted to be antigenic (through VaxiJen), non-allergenic (through AllergenFP and AllerTOP), and nontoxic were further considered [84,93]. These predictions were also cross-checked with experimentally known epitopes from databases like IEDB [89].

Using this strategy, different poultry pathogens were targeted. For H5N1, stem epitopes of the HA protein were identified as prospective broadly neutralizing agents due to their conservation across various clades [87]. The S1 domain of IBV displayed areas covering multiple serotypes, which are suitable for designing multivalent vaccines [78]. Epitopes identified within the cleavage activation site of the NDV F protein demonstrated considerable immunogenic potential across various virulent genotypes [79]. In the case of IBDV, loop areas within the VP2 protein were recognized and then cross-validated as pan-serotype epitopes using artificial intelligence-based cluster methods [95]. Additionally, epitopes from CAV VP1 and FPV 4b proteins were also recognized as structurally stable and conserved in field isolates [96]. The incorporation of AI tools has revolutionized target identification through a more scalable, reproducible, and adaptive process. AI facilitates a rational vaccine design beyond sequence conservation by including structural, immunological, and population-level characteristics to choose the most potent antigens for poultry pathogens.

### 3.3. The Procedure of Vaccine Design and Optimization Using the AI/ML Tools

Following the identification of the target proteins/immunogens, the process of vaccine design should be optimized. The ideal vaccine should ensure immunogenicity enhancement, along with assurance of a safe, stable, and effective formulation. This detailed process possesses AI contributions at every level [97].

The AI-driven tools assess the immunological properties of epitopes, as classification algorithms identify those exhibiting the greatest antigenicity, minimal allergenicity, and optimal HLA binding affinities [44]. For the optimization of multi-epitope constructs, the reverse vaccinology approach utilizes neural networks to incorporate data on virulence factors, proteomic signatures, and host immune responses [77].

For conceiving revolutionary peptide and mRNA sequences, advanced models such as generative adversarial networks (GANs) and reinforcement learning (RL) frameworks have been utilized. These AI systems yield vaccine constructs that are proven to be immunogenic, synthetically tenable, and thermodynamically sound [44]. They accomplish this without relinquishing these vital attributes. AI-powered codon optimization tools anticipate species-specific codon usage and enhance mRNA stability, thereby improving the translational efficiency of DNA and mRNA vaccines [98].

Artificial intelligence assumes a pivotal function. AI further eases adjuvant selection. Concerning synergistic adjuvant combinations, immune system modeling informs computational platforms that predict them. These models emulate interactions between vaccine constituents and pattern recognition receptors (PRRs) for adjuvant selection. These adjuvants maximize nature along with adaptive responses [77].

### 3.4. Roles of AI/ML in the Design and Development of the Next Generation Viral Vaccines for Common Viral Diseases of Birds

AI is preeminent in the domain that constitutes demographic extent. Mechanisms gauge predicted epitope representation across differing ethnic HLA haplotypes, thereby securing wide-ranging effectiveness plus diminishing vaccine failure via genetic variance.

AI has made the creation of vaccines combating novel and recurrent viral contagions easier. Throughout the COVID-19 pandemic, AI made the expeditious construction of mRNA vaccines easier. Deep learning was employed by companies such as Moderna as well as BioNTech for optimizing mRNA codon usage, identifying stable immunogenic regions, and analyzing spike protein sequences [98].

AI infrastructures have likewise helped vaccine development against diverse viral contagions. These include Nipah virus, Influenza D virus, and Rift Valley fever virus. Within these instances, AI models forecast possible zoonotic transmission, and this eased anticipatory vaccine formulation. Predictive models scrutinized viral genome sequences, mutation rates, and host receptor binding profiles to predict viral evolution and to select antigens [77].

AI has furnished vaccine design for animal viruses that are meaningful economically in veterinary medicine. Multi-epitope constructs were synthesized for viruses, including avian influenza, bovine coronavirus (BCoV), and porcine circovirus (PCV2). iNeo-Epp as well as VaxiJen denote instruments utilized within this endeavor. These vaccines were devised by deep learning frameworks while also forecasting immunodominant regions with filtering of potential cross-reactive or non-specific epitopes [77].

Additionally, AI conceived broad-spectrum vaccines once it discerned preserved sections throughout diverse viral lineages. These pan-epitope vaccines seek protection extensively. They hold importance especially for pathogens like influenza, including coronaviruses that undergo mutation swiftly [78].

### 3.5. Transforming the Preclinical and Clinical Vaccine Development Through the AI-Driven Simulations and Trial Optimization

Substantial preclinical trials are requisite for the conversion of AI-designed vaccines into clinical reality. Realization of clinical trials is likewise requisite. AI’s revolutionary function is clear in both domains [99].

Within preclinical research, AI-powered simulation platforms model immune responses in silico, and they predict cytokine profiles, antibody titers, plus T-cell activation. These simulations empower researchers to identify promising vaccine candidates prior to in vivo testing. This ascertainment curtails animal utilization and expedites schedules [44]. As an example, models of the digital immune system emulate human immune dynamics, offering a virtual testbed to assess vaccine constructs.

AI streamlines trial architecture along with subject assortment throughout clinical study formulation. It additionally streamlines terminal forecasting. Flexible study schematics rely upon forecasting simulation instruments. These tools scrutinize patient biomarkers, real-world evidence, and historical trial data. Platforms such as Trial Pathfinder employ natural language processing (NLP) and real-time analytics to pair individuals with appropriate trial arms for improving recruitment efficiency, plus data integrity [100].

The AI assists by categorizing trial populations based on genetic, demographic, and clinical data. This categorization ascertains depiction and improves mathematical validity [100]. Algorithms forecast dropout risks plus adherence likelihoods, which permits preemptive intervention.

The nascent implementation of AI within clinical research includes digital twins such as virtual patient models. Such models emulate singular reactions to vaccines, which eases customized forecasting of trials with posology strategies [99].

AI instruments further serve a function within post-marketing oversight since they scrutinize unfavorable incident submissions, social media outlets, and wellbeing archives for pinpointing safety signals plus effectiveness inclinations promptly [100].

## 4. Pipelines of Vaccine Design Using Enhanced Computational/AI/ML Tools

**Figure 2:**
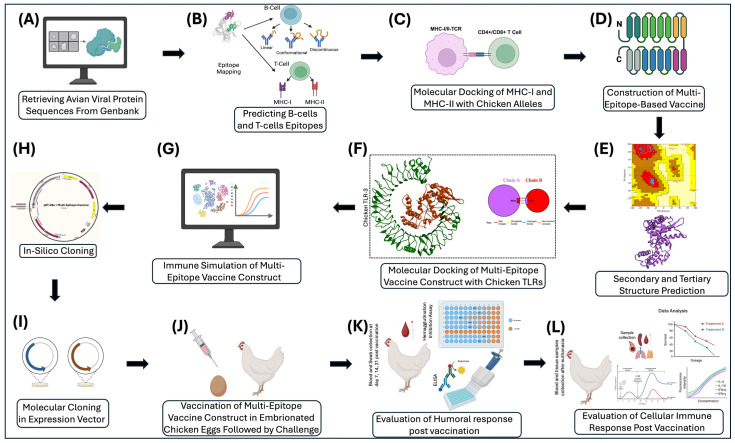
A schematic representation of the AI-driven workflow for designing and validating the next-generation vaccines, focusing on avian viruses to evaluate the efficacy of the vaccine on chickens and poultry. (**A**) Retrieval of avian viral proteins sequenced from GenBank. (**B**) Prediction of B-cell and T-cell epitopes from viral proteins. (**C**) Molecular docking of top-ranked MHC-I and MHC-II epitopes with chicken alleles. (**D**) Construction of the multi-epitope vaccine comprising selected B-cell, MHC-I, and MHC-II epitopes from viral proteins. (**E**) Secondary and tertiary structural modeling and validation of the multi-epitope vaccine construct. (**F**) Molecular docking of multi-epitope vaccine construct with chicken TLRs (TLR3), (**G**) In silico Immune simulation of the multi-epitope vaccine construct. (**H**) Codon optimization and in silico cloning of the multi-epitope vaccine construct into the pET-28a (+) expression vector. (**I**) Molecular cloning of the multi-epitope vaccine construct in a prokaryotic or eukaryotic expression vector. (**J**) Vaccination of embryonated chicken eggs at day 18, followed by viral challenge at 7–14 days of age. (**K**) Evaluation of humoral responses in chicken blood on day 7, 14, and 21 post challenge. (**L**) Comprehensive evaluation of cellular and humoral immune responses in trachea, spleen, lungs, thymus, blood, and swab samples after euthanasia at day 35–42. This figure was made using BioRender 2024.

A detailed schematic of the overall methods and procedures can be seen in Figure 2.

A schematic illustrates the overall methods and process for utilizing AI to create a vaccine for various prominent avian viruses. Target proteins will be identified for each virus, such as hemagglutinin in Avian Influenza, that have the potential to be vaccine targets. The sequence for each of the viral proteins will be retrieved from GenBank with their accession numbers documented. The sequences will be mapped to identify epitopes for both B-cells and T-cells. The highly immunogenic non-antigenic epitopes will be aligned and used to generate a mosaic vaccine. Molecular cloning in both prokaryotic and eukaryotic cells will be used to express viral vectors. To analyze binding, molecular docking will be used to check vaccine parameters. After virtually checking vaccine parameters, the vaccine will be simulated and used to stimulate the immune response. To fully understand the efficacy and safety of the vaccine, the vaccine will need to be tested in eggs and in chickens to test the immunogenicity of the vaccine.

### 4.1. Preprocessing and Curation of Viral Protein Sequences for the AI/ML-Based Vaccine Design for Some Common Viral Diseases of Poultry

The protein sequences of major poultry viral pathogens were systematically retrieved from publicly available databases, mainly NCBI GenBank [101] and UniProt [83], noting possible target proteins for vaccine development and the viral protein functions (Table 2). The selection criteria focused on economically important RNA viruses, namely the H5N1 avian influenza virus (AAR02643.1), infectious bronchitis virus (IBV), Newcastle disease virus (NDV), infectious bursal disease virus (IBDV), avian metapneumovirus, and DNA viruses like chicken anemia virus (CAV) and fowl pox virus (FPV) [78,80]. To avoid redundancy, CD-HIT clustering was applied at a 90% identity threshold to ensure dataset diversity while removing duplicates [102]. Multiple sequence alignment (MSA) was performed using Clustal Omega (version 1.2.4) [103] and MUSCLE [104] to assess sequence conservation, identify polymorphic regions, and facilitate epitope mapping. Alignment quality was manually inspected and adjusted to maintain biologically meaningful comparisons [105]. This preprocessing step ensured artificial intelligence models were trained on representative, high-quality sequence data that best represent the circulating viral strains.

### 4.2. Epitope Mapping and Antigen-Predicting Tools

The predictive identification of epitopes is a prerequisite to designing immunogenic vaccine candidates. The B-cell epitope prediction targeting both linear and conformational epitopes with several algorithms for improved accuracy [88]. For various avian viruses, linear epitopes were predicted using BepiPred 2.0, which utilizes a random forest machine learning method incorporating amino acid properties and structural features [105] (Table 3). Conformational epitopes, which are important in antibody binding, were predicted by DiscoTope 2.0, incorporating spatial information from 3D structures and amino acid statistics [119] (Table 3). For T-cell epitopes, NetMHCpan 4.1 and NetMHCIIpan 4.0 were employed in predicting peptide binding affinities to MHC class I and II alleles prevalent in Gallus gallus, specifically BF2 alleles that are well-defined in chickens (Table 3) [91]. Peptides with predicted half-maximal inhibitory concentration (IC50) values ≤ 50 nM were classed as high-affinity binders, which is suggestive of immunodominant potential [94]. Antigenicity was assessed using VaxiJen v2.0, an auto-cross covariance transformation-based protein sequence-to-uniform vector server, which distinguishes protective antigens from non-antigens without the need for sequence alignment [84] (Table 3). Utilization of a multi-tool strategy authenticated epitope selection, reducing false positives.

Table 3 summarizes representative epitopes identified from major poultry viral pathogens, including RNA viruses such as H5N1, IBV, NDV, IBDV, and Avian Leukosis Virus, as well as DNA viruses like Chicken Anemia Virus and Fowlpox Virus. For each epitope, we provide the corresponding viral protein, its function, the GenBank accession number, the predicted MHC binding affinity (IC50), the immunogenicity score, and the determined by VaxiJen v2.0. The epitope sequences were predicted using NetMHCpan 4.1, NetMHCIIpan 4.0, and BepiPred 2.0, selected based on criteria such as high binding affinity, antigenic potential, and sequence conservation. These epitopes serve as the foundation for designing multi-epitope vaccine constructs optimized for the MHC alleles of Gallus gallus.

The above epitope prediction tools were selected based on their accuracy and suitability in avian hosts. Bepipred helps in the prediction of linear B-cell epitopes with improved sensitivity. Due to its structural integrity and surface accessibility data, DiscoTope is used for conformational epitopes [124]. Using these tools together provides us with a robust framework for epitope mapping in avian systems.

### 4.3. Structural Modeling and Validation of Viral Proteins Using AlphaFold2 for Accurate Epitope Localization and Vaccine Design

**Figure 3:**
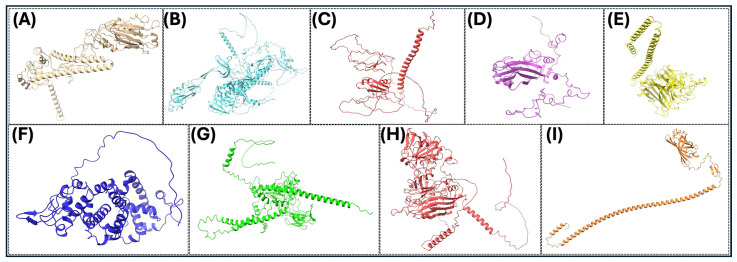
Three-dimensional (3D) protein structures of key viral proteins for some significant avian RNA viruses. (**A**–**I**) The 3D structures of the key viral proteins from the avian RNA viruses listed in Table 2 are shown. AlphaFold2 and Chimera X were utilized to generate the images. With these structures, they could be utilized to aid in vaccine design and development.

**Figure 4:**
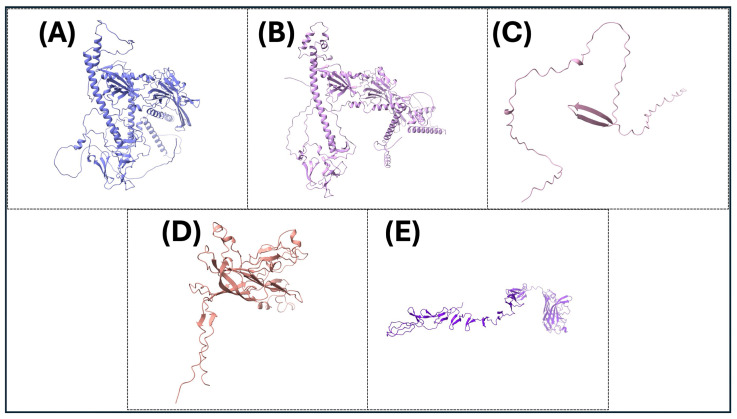
The three-dimensional (3D) protein structures of key viral proteins for some significant avian DNA viruses. (**A**–**E**) The 3D structures of the key viral proteins from the avian DNA viruses listed in Table 2 are shown. AlphaFold2 and Chimera X were utilized to generate images. With these structures, they could be utilized to aid in vaccine design and development.

The precise three-dimensional target protein models are critical to the localization of epitopes and the rational design of vaccines. The protein structures were predicted using AlphaFold2, an innovative deep learning protocol capable of predicting protein folds with near-experimental accuracy [85]. Sequences were deposited into the AlphaFold2 pipeline, which integrates multiple sequence alignments and pairwise residue information to generate full models [86]. The model confidence was assessed using per-residue predicted Local Distance Difference Test (pLDDT) scores, where scores above 70 were considered reliable [125]. The structural validation included analysis by using the Ramachandran plots using PROCHECK to assess backbone dihedral angles, in addition to ProSA-web for the analysis of overall model quality using z-score comparisons with experimentally determined structures. Structure visualization and analysis were performed using PyMOL (PyMOL is 3.1.6.1 (as of June 2025)) and UCSF Chimera (Version 1.17.3 (released July 2023)) to investigate epitope accessibility and surface mapping. The pipeline enabled the prediction of conformational B-cell epitopes with high accuracy and aided the selection of immunogenic areas based on structural parameters (Figure 3 and Figure 4).

### 4.4. Approaches to the Design and Optimization of Some Multi-Epitope-Based DNA Vaccine Constructs Against Some Common Avian Viruses

The epitope sequences that passed antigenicity and structural filters were concatenated into multi-epitope vaccine constructs by specific linkers to preserve epitope integrity and facilitate correct antigen processing. AAY linkers were used between cytotoxic T lymphocyte (CTL) epitopes to improve the efficiency of proteasomal cleavage, GPGPG linkers were used between helper T lymphocyte (HTL) epitopes to enhance the presentation of epitopes, and KK linkers separated B-cell epitopes to preserve flexibility To enhance immunogenicity, an N-terminal adjuvant sequence from chicken β-defensin and a universal T-helper epitope (PADRE) were added. The codon optimization of the complete construct sequence for expression in Gallus gallus was achieved using the JCat server [126], optimizing codon usage bias to enhance translation efficiency and mRNA stability. The physicochemical properties, like theoretical isoelectric point (pI), molecular weight, aliphatic index, grand average hydropathicity (GRAVY), and instability index, were calculated with ProtParam to assess construct stability and solubility.

### 4.5. The In Silico Assessment of the Immunogenicity, Allergenicity, Toxicity, and Structural Stability of the Multi-Epitope DNA Vaccine Constructs

For safety and effectiveness, vaccine construction underwent in silico immunogenicity and allergenicity evaluations. VaxiJen v2.0 was utilized again to validate overall antigenicity at a high threshold (>0.7) [84]. Allergenicity predictions were made using AllerTOP v2.0 and AllergenFP 1.0, classifying proteins according to auto-cross covariance transformation and physicochemical properties, respectively, to reduce possible allergic reactions [93], AllerTOP v.2—a server for the in-silico prediction of allergens. Peptide toxicity was predicted through ToxinPred, which forecasts toxic motifs and peptide toxicity based on Support Vector Machines trained on toxin databases. SOLpro predicted solubility upon expression to forecast the ease of recombinant protein production. Secondary structure was predicted with PSIPRED to evaluate the ratio of helices, sheets, and coils, towards vaccine stability.

### 4.6. In Silico Immune Simulation

The vaccine construct’s elicited immune responses were simulated using the C-ImmSim server, which models the mammalian and avian immune system dynamics by employing position-specific scoring matrices, cytokine networks, and cell population kinetics [92]. Three doses at four-week intervals were simulated to mimic the prime-boost vaccination regimen. Important parameters, such as antigen presentation, B-cell and T-cell population dynamics, cytokine release profiles (e.g., IFN-γ, IL-2), and antibody titters (IgM, IgG, IgA) were evaluated to predict the quality and duration of the immune response. The simulation provided useful information on the formation of memory B and T cells, helper T-cell polarization (Th1/Th2), and cytokine milieu, all of which are essential measures of vaccine efficacy. This in silico analysis enabled rapid and cost-effective screening of vaccine immunogenicity before laboratory validation.

To substantiate the in-silico vaccine construct, the vaccine constructs are to be cloned and expressed in E. coli or CHO cells, which confirms the yield, stability, and solubility of the protein expressed. To evaluate cellular immune responses, chicken PBMCs are isolated. For the in vivo evaluation, viral replication and antigenicity are to be measured in a standard embryonated SPF egg. To validate the efficacy of the vaccine construct, the immunized SPF chickens are then challenged with the target pathogen in terms of clinical signs, viral load, and histopathological analysis.

### 4.7. The AI/ML Parameters, Models, and Validation Techniques Used for the Design and Development of the Next-Generation Vaccines Against the Common Viral Diseases of Birds

A. Parameters of the epitope selection

We mainly rely on the structural stability of the selected B cell epitopes (linear/conformational). In the case of the T cell epitope selection, we mainly used the IC50 ≤ 50 nM as a high-affinity filter parameter. We also used the following parameters of Antigenicity (VaxiJen v2.0 > 0.7), non-allergenicity (AllerTOP v2.0, AllergenFP 1.0), and non-toxicity (ToxinPred) for further optimization of our epitope selection of the target vaccine. Finally, the selected/mapped epitopes should be cross validated against IEDB experimentally known epitopes.

B. Parameters related to the structural modeling and validation of the selected vaccines

The multiple sequence alignment for each target protein will be carried out, and the output data will be subjected to the alphafold analysis using the pLDDT scores (>70 = reliable). Further structural validation will be carried out using the Ramachandran plots (PROCHECK) and ProSA-web z-scores. This is in addition to the visualization of epitope accessibility using PyMOL/Chimera.

C. Parameters related to the vaccine design, construction of common viral diseases of birds

Using the appropriate linkers for the multiepitope vaccine for the common viral diseases of birds (AAY linkers (CTLs), GPGPG linkers (HTLs), KK linkers (B-cell epitopes)). The JCat, assessing GC content, Codon Adaptation Index (CAI), and mRNA stability, should be used to do codon optimization based on the Gallus gallus species.

D. Parameters related to the in silico immune simulation

We will use the C-ImmSim server with a prime-boost schedule (three doses, four-week intervals) as a standard protocol for the immune simulation of the designed vaccine.

E. Experimental validation parameters

Although the AI design and prediction are very powerful tools in vaccine design, laboratory/field validation using laboratory models and the actual host is mandatory to license any potential vaccines against the common viral diseases of birds. The validation will require testing of these vaccine constructs using (i) an in vitro system, using cell lines or immune cells. (ii) Ex vivo models, using some immune cells isolated from the spleen, bone marrow, or blood of the chickens. (iii) The in vivo studies are the most important key experiments to validate any vaccine to be licensed and fit for veterinary use against the target viral pathogens of interest. As per the USDA standards, the successful vaccines should offer at least 80% protection against the challenge of the wild-type virus of interest [127,128].

### 4.8. Validation/Cross-Validation Approaches for the Computational/AI/ML-Designed Vaccines for the Common Viral Diseases of Birds

Our approach, using AI on vaccine design and development, considers the cross-validation of our designed vaccines against some experimentally confirmed epitopes in IEDB, which serves as a benchmark database. We elaborated on the precise filtration parameters for each stem of the AI/ML vaccine pipelines above in Section 4.7.

### 4.9. The Integration of the Computational/ AI/ML Tools and Functional Validation to Enhance and Increase the Efficacy of the Next-Generation Vaccines Against the Common Viral Diseases of Birds

There is no doubt that the integration of the new computational tools, and various arms of the AI and the ML tools will enhance and speed up the process of vaccine design and development, but the experimental validation using the natural host is the milestone in the validation and licensing of any vaccine, not only for birds but also for human and other species of animals. The experimental validation is still also mandated by many government bodies that approve any vaccines, such as the FDA, USDA, and NIH.

## 5. Some Future Directions in the Application of AI/ML in Avian Viral Vaccine Design and Development

### 5.1. Integration of AI/ML and Other Bioinformatics Tools to Enhance the Vaccine Design and Development

The future of vaccine development is in the collaborative application of AI with other emerging technologies. For example, the combination of AI and synthetic biology allows for the design of new antigen constructs and delivery systems. AI-directed nanotechnology can promote targeted delivery of antigens and adjuvants with enhanced vaccine uptake and immune stimulation. The combination of CRISPR-mediated genome editing with AI has the potential to expedite vaccine target discovery by allowing large-scale functional validation [98].

### 5.2. Integration of AI/ML in the Personalized Vaccine Development

The ability of artificial intelligence to sift through high-dimensional patient data, including genomics, proteomics, and immune profiles, allows for the generation of personalized vaccines tailored to individual immune responses. Personalized cancer vaccines are already in clinical trials, where AI is used to identify tumor-specific neoantigens. Similar strategies are being used for infectious diseases, enabling AI to match vaccines to HLA profiles, past exposure history, and microbiome diversity. This shift in strategy has the promise of increasing efficacy while minimizing adverse events.

### 5.3. The Potential Roles of the AI Tools and Their Applications in the Emergency Preparedness Plans for the Next Pandemic

The genomic surveillance in real-time, combined with AI, can potentially identify and track mutations, forecast pathogen spread, and identify emerging zoonotic risks. AI can even simulate population immunity, optimize vaccine allocation, and redesign vaccine candidates dynamically according to viral mutation. Lessons from COVID-19 highlight the necessity of integrating AI into global health systems to reduce response time from years to weeks or even days.

## 6. Challenges and Limitations of the Use of AI/ML in Vaccine Design

### 6.1. Data Quality and Availability

The quality and availability of biological datasets are a significant concern for AI-driven vaccine development. To train accurate and generalizable models, AI algorithms need big, labeled datasets. But datasets about rare pathogens, neglected tropical diseases, or new zoonoses are often small, incomplete, or biased toward certain geographic or demographic groups. In addition, differences in sequencing standards and metadata annotation make the data even less useful. AI models may not work well with a wide range of people or animals if they do not have enough different data [77].

### 6.2. Some Ethical and Regulatory Challenges in the AI/ML-Driven Vaccine Development

AI-assisted health interventions make ethical problems even worse. Some of the problems are obtaining people’s permission to use their data, algorithmic bias, unequal access to AI-powered vaccines, and who owns AI-generated intellectual property. AI-designed vaccine candidates also have to go through rules that were not made for drugs that are made by computers [100]. The FDA and EMA are still working on standards for evaluating AI-driven drug and vaccine discovery, which could slow down the process of getting these drugs and vaccines into the clinic.

### 6.3. The Availability of the Infrastructure and Sustainability Challenges in Using AI/ML in Vaccine Development

The availability of computational resources, such as GPUs, high-performance cloud platforms, and safe data storage, is required for high-throughput AI models. Global disparities in vaccine innovation and access may worsen as a result of this requirement, which may restrict participation by institutions in low- and middle-income nations [97]. Large-scale model training also consumes a significant amount of energy and raises sustainability concerns, particularly in light of global climate goals.

## 7. Conclusions

The incorporation of artificial intelligence (AI) in vaccine development is a revolutionary step in the biomedical field, with unparalleled efficiency, accuracy, and flexibility. As presented in the course of this paper, AI can facilitate a shift from the data-oriented, predictive, and extremely personalized strategies. At the initial stage of vaccine development, AI greatly speeds up target identification and antigen discovery processes. Machine learning models and deep neural networks have shown their ability to mine high-throughput omics datasets, predict antigenic determinants, and model protein structures.

AI has made the generation of viral vaccines possible in both human and veterinary medicine in a remarkably short time. It allows for the generation of broad-spectrum and pan-strain vaccines through the identification of conserved viral areas and the prediction of viral evolution. In actual practice, AI-powered platforms have already helped in SARS-CoV-2 mRNA vaccine design, while the same strategies are being applied to develop vaccines against diseases like Nipah virus, Influenza D, and Rift Valley fever virus.

The intersection of AI with other technologies, including CRISPR, nanotechnology, and synthetic biology, will shape the next wave of vaccine innovation. AI-driven personalized vaccines based on genetic and immunological profiles are a new frontier in preventive medicine. Additionally, AI’s role in pandemic preparedness and real-time response highlights its essential contribution to global health security. Our lab focuses on AI/ML and in silico methods to generate compounds and vaccines for viruses such as H5N1, BCoV, and FIPV.

## Figures and Tables

**Table 1:** List of some selected viral vaccines developed using advanced Machine Learning and AI Technologies.

Virus	Host	AI Method	Reference
Nipah Virus	Animal Reservoirs	Epitope mapping, structural validation	[20]
Machupo Virus	Animal Reservoirs	Epitope prediction, vaccine construct design	[21]
H5N1 Influenza	Birds	B-/T-cell epitope pipeline + docking	[22]
MVEV	Birds/Mammals	Envelope epitope mapping, structural validation	[23]
Feline Infectious Peritonitis Virus (FIPV)	Cats	Epitope prediction, docking	[8,24]
BCoV	Cattle	ML epitope mapping + AlphaFold2	[5]
Foot-and-Mouth Disease Virus (FMDV)	Cattle	Immunoinformatics, structural modeling	[25]
Foot-and-Mouth Disease Virus (FMDV)	Cattle	Epitope fusion with TLR agonist	[26]
FMDV	Cattle	Multi-serotype epitope design, stability/autogenetics analysis	[27]
FMDV	Cattle	Serotype mapping, structural validation	[25]
BEFV	Cattle	Multi-epitope subunit	[28]
Bovine Leukemia Virus (BLV)	Cattle	Epitope prediction, MD, immune simulation	[29]
Lumpy Skin Disease Virus (LSDV)	Cattle	Epitope prediction, TLR docking, MD simulation	[30]
Newcastle Disease Virus (NDV)	Chickens	ANN epitope affinity prediction, docking	[31]
Avian Leukosis Virus (ALV)	Chickens	Peptide design, TLR7 docking	[32]
Infectious Bursal Disease Virus (IBDV)	Chickens	Immunoinformatic (VP2/VP3 proteins)	[33]
CPV-2	Dogs	Immunoinformatics + docking	[34]
Canine Circovirus (CanineCV)	Dogs	Immunoinformatics, in vivo validation	[35]
Rota virus	Elephants	Epitope prediction, structural validation, and immune simulation	[36]
Goatpox Virus (GTPV)	Goats	Epitope mapping, TLR docking, immune sim	[37]
Orf Virus	Goats/Sheep	Immunoinformatics, epitope selection	[38]
Zika	Humans	Epitope prediction + docking	[39]
Dengue (multi-serotype)	Humans	Structural modeling + conserved targeting	[40]
Rift Valley Fever Virus (RVFV)	Livestock	Epitope prediction, docking, and molecular dynamics	[41]
Influenza D Virus (IDV)	Livestock	Epitope mapping, allergenicity & toxicity checks	[42]
Hendra Virus (HeV)	Livestock	Epitope prediction, immune simulation, TLR docking	[43]
HeV	Livestock	Epitope screening, TLR docking	[44]
Marburg Virus (MARV)	Non-human primates/Humans	Reverse vaccinology, epitope mapping, TLR docking	[45]
ASFV	Pigs	Immunoinformatics + docking + simulation	[46]
Porcine Epidemic Diarrhea Virus (PEDV)	Pigs	Epitope selection, TLR docking, immune sim	[47]
Porcine Epidemic Diarrhea Virus (PEDV)	Pigs	Epitope selection, immune simulation	[48]
PRRSV	Pigs	Conserved epitope selection, immune modeling	[49]
Avian Influenza A (H5N1)	Poultry	Reverse vaccinology, epitope mapping	[6,22]
RVFV	Ruminants	ML peptide prediction + immune simulation	[41]
RVFV (M-protein)	Ruminants	Reverse vaccinology + docking	[50]
Bluetongue Virus (BTV)	Sheep	Epitope mapping, TLR docking	[51]
ASFV	Swine	Reverse vaccinology + dynamics	[46]
African Swine Fever Virus (ASFV)	Swine	Reverse vaccinology, SLA docking, MD simulations	[52]
Porcine Reproductive and Respiratory Syndrome Virus (PRRSV)	Swine	Epitope mining, antigenicity/allergenicity	[53]
ASFV	Swine	Reverse vaccinology, SLA docking, MD simulations	[54]
PRRSV	Swine	Global epitope mining, antigenicity/allergenicity profiling	[49]
ASFV	Swine	SLA docking, MD simulation	[46]
Poxviruses	various	Proteome-wide AI epitope predictor	[55]

**Table 2:** Information on some key target viral proteins that could be used for vaccine development of some viral diseases of poultry.

Serial Numbers	RNA Viruses	Target Protein	Protein Function	Protein Length (aa)	Structural Coverage by Alpha Fold 2	Accession Numbers of the Reference NCBI Sequences for Each Virus	References
1.	Avian Influenza	HA	Binds to host cell receptors (sialic acids)	1760	81	NC_007362.1	[106]
2.	Infectious Bronchitis Virus (IBV)	Spike	Inserts into the ligand on the surface of the host cell receptor, opening the cell wall	3461	58	NC_048213.1	[107]
3.	Avian Metapneumovirus	G protein	Attachment to the host cell surface receptor allows viral entry into the host	1175	0	NC_039231.1	[108]
4.	Avian Encephalomyelitis	VP1	Host protective immunogen	270	63	AFM73888.1—VP1 partial	[109]
5.	Newcastle Disease	HN	Bind to host cell receptors	1715	81.4	NC_039223.1	[110]
6.	Avian Bornavirus	N and P protein	Packing viral RNA, essential for polymerase activity, shuttles RNP into and out of the nucleus; a cofactor of bornavirus polymerase	1121, 605	64.2	NC_039189.1	[111]
7.	Avian Leukosis	G protein	Mediates viral attachment to the cell surface	2111	53.3	MT179556.1	[112]
8.	Avian Reovirus	Sigma C	binding to the host cell surface	326	71.4	AAK18188.1	[113]
	**DNA Viruses**	**Target Protein**	**Protein Function**	**Protein Length (bp)**	**Structural Coverage by Alpha Fold 2**	**Accession Numbers**	**References**
9.	Infectious Laryngotracheitis Virus	B-glycoprotein spike	viral attachment to the host cell surface to form a heterodimer	2651	62.7	NC_006623.1	[114]
10.	Marek’s Disease (Gallid Herpesvirus-2)	B-glycoprotein spike	viral attachment to the host cell surface to form a heterodimer	2597	62.8	NC_002229.3	[115]
11.	Infectious Anemia Virus	VP3	induces apoptosis in chicken lymphocytes	388	29.8	AF199501.1	[116]
12.	Avian Polyoma Virus	VP1	Capsid protein that binds to host cell receptors for infection	1031	87.2	PP057981.1	[117]
13.	Fowl Adenovirus	Fiber Genes	responsible for hemagglutination	1386	81.3	DQ864436.1	[118]

**Table 3:** Identification and characterization of some predicted B- and T-Cell epitopes for the multi-epitope vaccine design for some viral diseases of poultry.

Types of Epitopes	Recognized by	Immune Function	AI Prediction Tool Used	Reference
Linear B Cell Epitopes	B-cell Receptors (BCRs)	Induce antibody production; direct neutralization of extracellular virus	BepiPred 2.0, ABC Pred	https://services.healthtech.dtu.dk/services/BepiPred-2.0/ (accessed on 14 September 2025) [120]
Conformational B-cell Epitopes	BCRs (3D-dependent)	Target protein folding-dependent antigenic sites	DiscoTope, Ellipro	https://services.healthtech.dtu.dk/services/DiscoTope-3.0/ (accessed on 14 September 2025) [121]
CD8+ T cell Epitopes	Cytotoxic T Lymphocytes (CTLs)	Kill infected cells by recognizing MHC-I-bound peptides	NetMHCpan	https://services.healthtech.dtu.dk/services/NetMHCpan-4.1/ (accessed on 14 September 2025) [122]
CD4+ T cell Epitopes	Helper T Lymphocytes (Th cells)	Aid in inactivating B cells and CTLs via cytokine release	NetMHCIIpan	https://services.healthtech.dtu.dk/services/NetMHCIIpan-4.0/ (accessed on 14 September 2025) [122,123]

## Data Availability

No new data were created or analyzed in this study. Data sharing is not applicable to this article.

## Abbreviations

The following abbreviations are used in this manuscript:

AI	Artificial intelligence
ML	Machine learning
DNA	Deoxyribonucleic acid
RNA	Ribonucleic acid
SARS-CoV-2	severe acute respiratory syndrome coronavirus-2
COVID-19	Coronavirus disease-2019
USDA	United States Department of Agriculture
FDA	Food and Drug Administration
